# A New Method to Measure Portal Venous and Hepatic Arterial Blood Flow Patients Intraoperatively

**DOI:** 10.1155/1996/15760

**Published:** 1996

**Authors:** F. Jakab, Z. Ráth, F. Schmal, P. Nagy, J. Faller

**Affiliations:** Department of Surgery Semmelweis University of Medicine & St. John Hospital Budapest Hungary Supported by a grant from Ministry of Welfare M-005/1990 Hungary

## Abstract

The intraoperative measurement of the afferent circulation of the liver, namely the hepatic artery flow and portal venous flow was carried out upon 14 anesthetized patients having carcinoma in the splanchnic area, mainly in the head of the pancreas by means of transit time ultrasonic volume flowmeter. The hepatic artery flow, portal venous flow and total hepatic flow were 0.377±0.10; 0.614±0.21; 0.992±0.276 l/min respectively.

The ratio of hepatic arterical flow to portal venous flow was 0.66±0.259 There was a sharp, significant increase in hepatic arterial flow (29.8±6.1%, p<0,01) after the temporary occlusion of the portal vein, while the temporary occlusion of hepatic artery did not have any significant effect on portal venous circulation. The interaction between hepatic arterial flow and portal venous flow is a much disputed question, but according to the presented data here, it is unquestionable, that the decrease of portal venous flow immediately results a significant increase in hepatic artery circulation.


*HPB Surgery*, 1996, Vol.9, pp.239–243
Reprints available directly from the publisher
Photocopying permitted by license only

© 1996 OPA (Overseas Publishers Association)
Amsterdam B.V. Published in The Netherlands
by Harwood Academic Publishers GmbH
Printed in Malaysia

### A New Method to Measure Portal Venous and Hepatic Arterial Blood Flow in Patients Intraoperatively

F. JAKAB, Z. RÁTH, F. SCHMAL, P. NAGY and J. FALLER

Department of Surgery, Semmelweis University of Medicine & St. John Hospital Budapest, Hungary
Supported by a grant from Ministry of Welfare, HUNGARY M-005/1990

*(Received 23 October 1993)*

The intraoperative measurement of the afferent circulation of the liver, namely the hepatic artery flow and portal venous flow was carried out upon 14 anesthetized patients having carcinoma in the splanchnic area, mainly in the head of the pancreas by means of transit time ultrasonic volume flowmeter. The hepatic artery flow, portal venous flow and total hepatic flow were 0.377±0.10; 0.614±0.21; 0.992±0.276 1/min respectively.

The ratio of hepatic arterical flow to portal venous flow was 0.66±0.259

There was a sharp, significant increase in hepatic arterial flow (29.8±6.1%, p<0,01) after the temporary occlusion of the portal vein, while the temporary occlusion of hepatic artery did not have any significant effect on portal venous circulation. The interaction between hepatic arterial flow and portal venous flow is a much disputed question, but according to the presented data here, it is unquestionable, that the decrease of portal venous flow immediately results a significant increase in hepatic artery circulation.

KEY WORDS: Transit time ultrasonic volume flowmetry hepatic artery flow portal venous flow afferent circulation of liver

Numerous methods are known for the measurement of hepatic blood flow in experimental animals and humans, but most techniques have several weak points[2]. In clinical practice the most commonly used method for the measurement of afferent circulation of the liver is an electromagnetic flowmeter. The data gained by the electromagnetic flowmeter indicate, that the measurement of portal venous flow may not be precise[9]. In addition the extensive preparation of the vessels restricts clinical usefulness of the electromagnetic flowmeter in humans. In experimental condition accuracy of venous flow measurements can be achieved by the repeated measurements[10,11].

Transit time ultrasonic volume flowmeter has recently been introduced to experimental and later on to clinical practice and the measurement of arterial and venous blood flow has been reported in experimental animals with high reproducibility and stability[1].

However, measurement of splanchnic blood flow, the afferent circulation of the liver using transit time ultrasonic volume flowmeter in patients has been reported by only one group of workers in this field[3].

The data from simultaneous measurements of hepatic artery (HA) and portal vein (PV) by means of transit time ultrasonic volume flowmeter in patients who underwent abdominal operations are presented herein.

*Correspondence to*: Ferenc Jakab M.D.Ph.D. Professor of Surgery Budapest, Uzsoki street 2p. HUNGARY, H-1145 Fax/Phone: 36-1 156-3049

### MATERIALS AND METHODS

Fourteen patients with a mean age of 51,5 years (a range of 37 to 71 years) underwent abdominal interventions for carcinoma of the splanchnic area, mainly carcinoma of the head of the pancreas. The informed consent had been obtained from all patients.

The abdominal interventions were carried out under general anesthesia with endotracheal intubation.

The blood pressure, blood pH, $O_2$ saturation were monitored carefully, and adequate fluid restoration was administered to prevent a drop in blood pressure. Only those patients were included in the study, were the changes of blood pressure did not exceed 10%.

The flow probes were placed on the HA and the PV after the isolation of these vessels. The measurement were carried out mainly during pancreato duo denectomy for carcinoma of the head of the pancreas and these vessels were isolated for the block dissection.

The simultaneous measurements of hepatic artery flow (HAF) and portal venous flow (PVF) were obtained by means of a transit time ultrasonic volume flowmeter (Model HT 207, Transonic System, inc. Ithaca, N.Y., U.S.A.)

After registration of the basal values of HAF and PVF, the occlusion of PV was carried out with fingers as the most atraumatic method. The occlusion-time lasted for 60 seconds. After the restoration of the afferent circulation of the liver, the temporary occlusion of proper hepatic artery was performed to clarify if that would affect PVF.

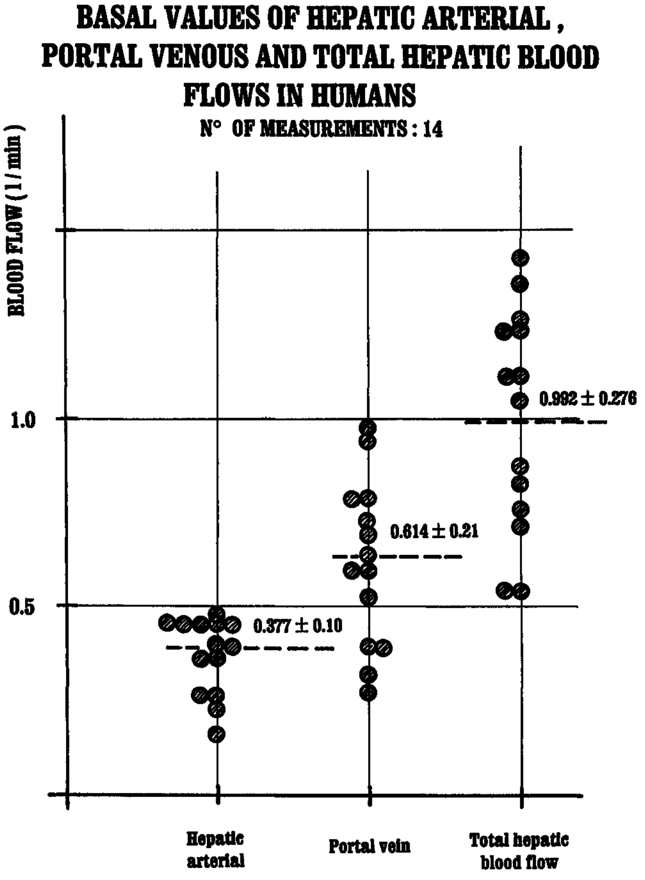

**Figure 1** Basal values of hepatic arterial (HAF), portal venous (PVF) and total hepatic blood flows (THBF). The horisontal lines represent the means, ± is SE.

*Abbreviations:* HAF: hepatic arterial flow, PVF: portal venous flow, THBF: total hepatic blood flow, HA: hepatic artery, PV: portal vein

The values of HAF and PVF were recorded continuously, Data were expressed as a mean, plus or minus one standard error. (SE) , Student's test was used. A 5 percent level of significance was chosen. For bio metrical analysis the Mattheas programme was applied.

### RESULTS

The basal values of HAF and PVF are illustrated in Figure 1. The basic HAF 0.377±0.10 l/min in average, while the PVF was measured as 0.614±0.21 l/min respectively. The basal HAF and PVF per body weight were 4.95±0.55 ml/min/kg and 12.9±1.1 ml/min/kg.

The total hepatic blood flow/THBF/was 0.992 l/min or 17.6±1.5 ml/min/kg.

The ratios of HAF to PVF, HAF to THBF and PVF to THBF are shown in Figure 2. and there values are as follows: 0.66±0.259 0.38±0.084, 0.61±0.085 respectively.

The temporary occlusion of portal vein immediately resulted in a sudden, and significant increase of HAF ($p < 0.01$). The rate of increase in HAF was 29.8±6.1% of the basal flow (Figure 3). The occlusion of HA did not alter the PVF significantly (Figure 4).

### DISCUSSION

The importance of the quantitative measurement and evaluation of the afferent circulation and perfusion of the liver is clear. The data gained under varying pathologic conditions can have a wide range of the

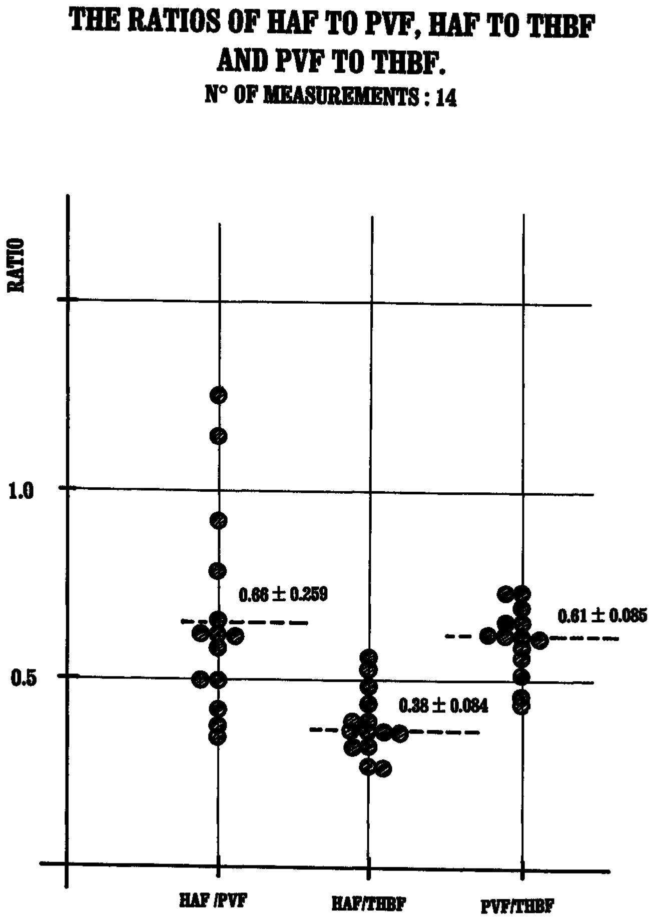

**Figure 2** The ratios of hepatic arterial flow to portal venous flow, (HAF/PVF), hepatic arterial flow to total hepatic blood flow (HAF/THBF) and portal venous flow to total hepatic flow (PVF/THBF) The horisontal lines represent the means, ± is SE.

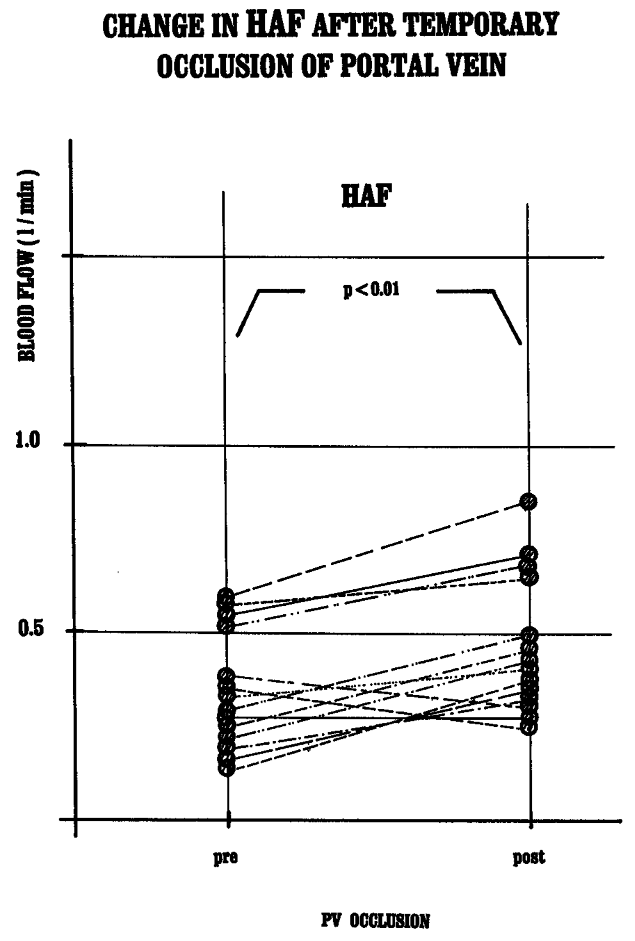

**Figure 3** The effect of temporary occlusion of portal vein (PV) on hepatic arterial flow (HAF).

diagnostic and therapeutic implications. In surgical practice the exact hemodymamic data regarding the liver can have a great effect on the choice of a particular operative interventions, such as extensive hepatectomy or porta systemic shunting. For this reason many different techniques have been employed in attempts to measure the hepatic blood flow in man. The earliest workers approached the problem using direct techniques which had no application in clinical investigations. Subsequent developments allowed an indirect determination of blood flow by the use of a variety of indicator clearence techniques which could also be applied to the clinical situation, but with diminished accuracy in the presence of liver disease.

Further shortcomings of the indirect methods are the inability to separate portal venous flow from hepatic arterial flow and inability to respond to rapid alteration in flow rate.

Although the electromagnetic flowmeter can separate PVF from HAF by direct measurement, this technique needs extended exposure and preparation of the afferent vessels (HA, PV) and the perfect fitting of the flowprobes. These disadvantages of the method restrict the clinical use of the electromagnetic flowmeter, on the other hand the new device for blood flow measurements, that is transit time ultrasonic flowmeter can be applied easily and quikly.

The greatest advantage of the method, that the flow probes require only acoustic contact and a loose fit gives perfect measurements. Long preparation is not necessary and as for ourselves we could start the blood flow measurements within 5 minutes, and the whole procedure did not exceed 12–13 minutes.

In the literature there are only a few reports on liver circulation in anesthetized patients during surgical intervention.[3,8,9] The THBF was a mean of approximately 1000ml/min in cholecystectomised patients, and these data closely correlate with our results, according to our measurements the THBF as 0.998 l/min in average[9]. According to Doi and his cooworkers the THBF proved to be 1010±52,7 ml/min in patient during surgical procedures under general anesthesia. These results also correlate with our measurements [3].

The ratio of HAF/PVF and HAF/THVF shows great variation according to our observations and the literature [3,8,9].

The interaction between the HAF and PVF is a much dipute question. In the present study it is clearly demonstrated that the occlusion of a PV, that is the decrease of PVF results in a significant, sudden increase of HAF. Most probably the temporary occlusion of PV, the reduced portal inflow has effect on intrahepatic distribution of portal and hepatic arterial blood flows, the hepatic arterial resistance decreases so that the hepatic arterial flow (HAF) increases [4,5,6,7]. The occlusion of HA did not have any effect on PVF suggesting the portal venous flow is mainly dependent on the circulation of the splanchnic area[5].

The study of the afferent circulation of the liver by means of transit time ultrasonic volume flowmetry seems to have great pathophysiological and clinical importance. The clinical application can cover the field of liver tumours, portal hypertension and pancreatic diseases.

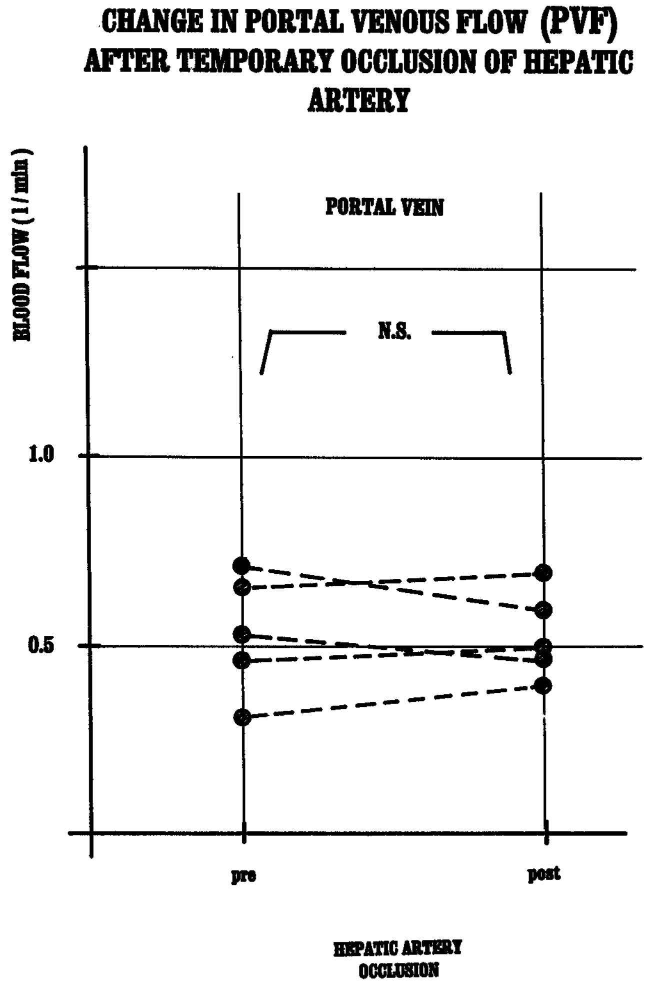

**Figure 4** The effect of temporary occlusion of hepatic artery (HA) on portal venous flow (PVF). N.S.: not significant.

### REFERENCES

1. Barnes R. J., Comline., R. S., Dobson A., and Drost, C. J.: (1983).An implantable transit time ultrasonic bloodflow meter *J. Physiol.* **345**: 2–3.
2. Bradley III. E. L.: (1974) Measurements of hepatic blood flow in man. Surgery. **75**: 783–789.
3. Doi, R., Inove K., Kogire M. (1988) *et al*: Simultaneons measurement of hepatic arterial and portal venous flows by transit time ultrasonic volume flowmetry. Surg. Gynecol. Obstet. **167**: 65–69.
4. Greenway C. V., Stark R. D. (1971) Vascular Bed. Physiological Rewiew **51**: 23–65.
5. Greenway, C. V., Oshiro. G: (1972). Intrahepatic distribution of portal and hepatic arterial blood flows in unanesthetized cats and dogs and the effects of portal occlusion, raised venous pressure and histamine. *J. Physiol.* **227**: 475–485.
6. Mathie R. T., Lewis M. H., Blumgart L. H.: (1988) Hepatic Hemodynamics after chronic Obstruction on the Biliary Tract in the Dog. *Surg. Gyn. Obstet.* **166**: 125–130.
7. Nagorney D. M., Mathie R. T., Lygidakis N. J., Blumgart L.H: (1982). Bile duct pressure as a modulator of liver blood flow after common bile duct obstruction. *Surg. Forum.* **33**: 206–208.
8. Payen D. M., Fratacci M. D., P. Dupuy (1990) *et al*: Portal and hepatic arterial blood flow measurements of human transplanted liver by implanted Doppler probs. *Surgery.* **107**: 417–427.
9. Schenk W. G., Mc. Donald J. C., Mc. Donald K., Drapanas T. (1962): Direct measurement of hepatic blood flow in surgical patients: with related observations on hepatic flow dynamics in experimental animals. *Ann. Surg.* **156**: 463–469.
10. Szabó G., Jakab F., Magyar Z.: (1974) Effect acutecholestasis on hepatic circulation *Acta. Med. Acad.Sci. Hung.* **31**: 229–239.
11. Szabó G., Jakab F., Magyar Z.: (1974). The mechanism of the effect of increased biliary pressure on hepatic circulation. *Acta Med. Acad. Sci. Hung.* **31**: 241–250.

